# Rice Weevil (*Sitophilus oryzae* L.) Gut Bacteria Inhibit Growth of *Aspergillus flavus* and Degrade Aflatoxin B1

**DOI:** 10.3390/jof10060377

**Published:** 2024-05-24

**Authors:** Haneen Abdullah Al-Saadi, Abdullah Mohammed Al-Sadi, Ali Al-Wahaibi, Ali Al-Raeesi, Mohamed Al-Kindi, Sathish Babu Soundra Pandian, Majida Mohammed Ali Al-Harrasi, Issa Hashil Al-Mahmooli, Rethinasamy Velazhahan

**Affiliations:** 1 Department of Plant Sciences, College of Agricultural and Marine Sciences, Sultan Qaboos University, Al-Khoud, Muscat 123, Oman; s110439@student.squ.edu.om (H.A.A.-S.); alsadi@squ.edu.om (A.M.A.-S.); awahaibi@squ.edu.om (A.A.-W.); ali.raeesi@gmail.com (A.A.-R.); mageda502@gmail.com (M.M.A.A.-H.); issah@squ.edu.om (I.H.A.-M.); 2 College of Medicine and Health Sciences, Sultan Qaboos University, Al-Khoud, Muscat 123, Oman; alkindi2@squ.edu.om; 3 Central Analytical and Applied Research Unit, Sultan Qaboos University, Al-Khoud, Muscat 123, Oman; sathish@squ.edu.om

**Keywords:** aflatoxin B1, antifungal, biological degradation, degradation products, mycotoxin, volatile organic compounds

## Abstract

In this study, bacteria residing in the gut of the rice weevils (*Sitophilus oryzae* L.) (Coleoptera: Curculionidae) feeding on aflatoxin-contaminated corn kernels were isolated and evaluated for their ability to suppress *Aspergillus flavus* and to remove/degrade aflatoxin B1 (AFB1). Four morphologically distinct *S. oryzae* gut-associated bacterial isolates were isolated and identified as *Bacillus subtilis* (RWGB1), *Bacillus oceanisediminis* (RWGB2), *Bacillus firmus* (RWGB3), and *Pseudomonas aeruginosa* (RWGB4) based on 16S rRNA gene sequence analysis. These bacterial isolates inhibited *A. flavus* growth in the dual culture assay and induced morphological deformities in the fungal hyphae, as confirmed by scanning electron microscopy. All four bacterial isolates were capable of removing AFB1 from the nutrient broth medium. In addition, culture supernatants of these bacterial isolates degraded AFB1, and the degradation of toxin molecules was confirmed by liquid chromatography-mass spectrometry. The bacterial isolates, *B. subtilis* RWGB1, *B. oceanisediminis* RWGB2, and *P. aeruginosa* RWGB4, were capable of producing antifungal volatile organic compounds that inhibited *A. flavus* growth. These results suggest that the bacterial isolates from *S. oryzae* gut have the potential to bind and/or degrade AFB1. Further research on their application in the food and feed industries could enhance the safety of food and feed production.

## 1. Introduction

Aflatoxin contamination in foods, as well as in livestock and poultry feeds, is a major global concern due to its carcinogenic and immunosuppressive properties [1,2]. The ingestion of an aflatoxin-contaminated diet can lead to acute and chronic aflatoxicosis in both humans and animals. Chronic aflatoxicosis can result in hepatocellular carcinoma, cirrhosis, and impaired immunity. Acute aflatoxicosis is characterized by symptoms like high fever, vomiting, liver failure, ascites, edema of feet, and jaundice. Prolonged exposure to aflatoxins may increase the risk of liver, lung, kidney, or colon cancers in both humans and animals [3]. Furthermore, pulmonary aspergillosis has been reported to aggravate the severity of coronavirus disease in immunocompromised individuals [4]. Twenty-eight fungal species belonging to *Aspergillus* sections *Flavi* (22 species), *Nidulantes* (4 species), and *Ochraceorosei* (2 species) have been identified as aflatoxin producers [5]. The primary producers of aflatoxins are *Aspergillus flavus*, *A. parasiticus*, and *A. nomius* [5,6]. Aflatoxin is produced and stored in specialized vesicles called “aflatoxisomes” within toxigenic cells of *Aspergillus* spp. before being exported by exocytosis [7]. A wide variety of agricultural commodities, including corn, peanut, sorghum, rice, pearl millet, soybean, sunflower, cotton, chili, black pepper, pistachio, almond, Brazil nut, walnut, and coconut are commonly contaminated with aflatoxins [8]. 

Aflatoxins are chemically classified as difurocoumarin derivatives, consisting of a bifuran ring fused to a coumarin nucleus, with a pentenone ring in B-type aflatoxins or a lactone ring in G-type aflatoxins. Currently, more than 20 types of aflatoxins have been identified [9]. Among them, aflatoxin B1 (C_17_H_12_O_6_; MW 312), B2 (C_17_H_14_O_6_; MW 314), G1 (C_17_H_12_O_7_; MW 328), and G2 (C_17_H_14_O_7_; MW 330) are frequently found in food commodities [3,10]. Aflatoxin M1 (C_17_H_12_O_7_; MW 328) and M2 (C_17_H_14_O_7_; MW 330) are metabolites of aflatoxins B1 and B2, respectively, commonly found in the milk of lactating mammals fed with aflatoxin-contaminated feed [6]. Aflatoxin B1 (AFB1; PubChem CID: 186907) is the most hazardous foodborne mycotoxin to humans and animals, classified by the International Agency for Research on Cancer as a Group1 carcinogen [11]. 

Various detoxification methods have been used to mitigate aflatoxin contamination in foods and feeds, with the goal of minimizing their deleterious effects [12]. Physical degradation methods encompass heat treatment, ultraviolet irradiation, Cobalt-60 gamma irradiation, photocatalysis, electron beam irradiation, and cold plasma treatment. Chemical degradation methods include ozonation, chlorine dioxide oxidation, and treatments with lactic acid, while biological degradation utilizes beneficial microorganisms and natural plant products [13]. Of the various techniques, biological degradation methods are considered efficient and environmentally friendly [14,15,16]. Biologically based approaches primarily focus on suppressing the growth of toxigenic *Aspergillus* spp. and detoxifying aflatoxin-contaminated agricultural products [17]. Several strains of lactic acid bacteria and yeast can bind to mycotoxins and reduce their bioavailability in foods or feeds [14,18,19]. In addition, the microbial enzymes can transform mycotoxins, further reducing their toxicity before excretion [20]. Different types of bacteria found in the intestinal tract of animals [21,22,23], soil, and other environments [24,25,26,27] have been reported to detoxify mycotoxins. Insects, in particular, stored product insects, are commonly exposed to aflatoxins in their natural environment. These insects are known to possess detoxification mechanisms that enable the breakdown and elimination of toxins from their system [28,29]. The involvement of metabolic enzymes such as NADPH-dependent reductases and hydroxylases in the detoxification of aflatoxins in insects has been documented [29]. Diverse microorganisms, including fungi, bacteria, protists, and archaea, have been found to live in insect guts [30,31]. Various factors, including gut anatomy, digestive enzymes, pH, and diet, influence the diversity of the gut microbiota [30,32]. The gut microbial communities play important roles in feeding, digestion, protection from pathogens and parasites, and resistance to pesticides [31]. We hypothesized that the symbiotic bacteria living in the gut of insects may possess the capacity to bind/degrade AFB1 and reduce its toxic effects. The objectives of this study were to (i) isolate and characterize bacteria residing in the gut of the rice weevil (*Sitophilus oryzae* L.) (Coleoptera: Curculionidae) collected from AFB1-contaminated corn kernels, (ii) assess in vitro antagonistic potential of bacterial isolates against *A. flavus*, (iii) determine AFB1 degradation ability of the bacterial isolates and their culture supernatants, (iv) analyze the degradation products of AFB1 following treatment with the culture supernatants of the bacterial isolates, and (v) test the potential of the bacterial isolates to produce antifungal volatile organic compounds (VOCs) against *A. flavus*.

## 2. Materials and Methods

### 2.1. Collection of Rice Weevils

Adult rice weevils feeding on corn kernels contaminated with over 20 ppb of AFB1 were collected from the Department of Plant Sciences, Sultan Qaboos University, and processed immediately. 

### 2.2. Molecular Identification of Rice Weevil

To confirm the identity of the weevil used in this study, molecular analysis was conducted on it. The weevil’s head and thorax were aseptically dissected, and genomic DNA was isolated using a modified CTAB protocol [33]. The mitochondrial cytochrome oxidase I (COI) gene of the weevil was amplified by PCR with LCO (5′-GGTCAACAAATCATAAAGATATTGG-3′) as the forward primer and HCO (5′-TAAACTTCAGGGTGACCAAAAAATCA-3′) as the reverse primer [34]. The PCR was carried out in a Veriti 96-well Thermal cycler (Applied Biosystems, Singapore) using a reaction volume of 25 µL containing 2 µL of DNA (50 ng), 1 µL of each primer (20 pmol) and a PuReTaq Ready-To-Go PCR Bead (Cytiva, Global Life Sciences Solutions Operations UK Ltd., Little Chalfont, Buckinghamshire, UK) dissolved in 21 µL of sterile distilled water (SDW). The PCR cycles and conditions were according to Abbasi et al. [35]. The sequence of the PCR product was determined (Macrogen Inc., Gangnam-gu, Seoul, Republic of Korea), and the nucleotide sequences were compared with reference sequences in the GenBank (http://www.ncbi.nlm.nih.gov; accessed on 30 October 2023) using the BLASTN tool.

### 2.3. Isolation of Rice Weevil Gut Bacteria

The adult weevils were starved overnight before the experiment. Each weevil was surface sterilized by immersing in 70% ethanol for 1 min and rinsed with SDW. The dissection was performed in a laminar flow hood using a microscope, sterile forceps, and fine needles. The entire gut was dissected and transferred to a sterile 1.5 mL microcentrifuge tube containing 300 µL of SDW and then ground using a sterile micropestle. The homogenate was streaked onto nutrient agar (NA; Oxoid Ltd., Basingstoke, Hampshire, UK) media using a sterile inoculation loop and incubated at 30 °C for 48 h. After incubation, the plates were examined for bacterial growth, and colonies differing in morphology were selected. Pure cultures of the bacterial isolates were obtained by the streak plate method.

### 2.4. Identification of Rice Weevil Gut Bacteria

The identity of the bacterial isolates from the weevil gut was determined on the basis of 16S rRNA gene sequence analysis. Each bacterial isolate was cultured in 100 mL of nutrient broth (NB) at 27 °C for 48 h with continuous shaking. DNA was isolated from the bacteria using a food-proof StarPrep Two kit (BIOTECON Diagnostics GmbH, Hermannswerder, Potsdam, Germany) following the manufacturer’s instructions. The extracted DNA was used as a template for PCR. A part of the bacterial 16S rRNA gene was amplified using the universal 27F and 1429R primers [36] and PuReTaq Ready-To-Go PCR beads, as described by Al-Hussini et al. [37]. The PCR products were sequenced (Macrogen Inc., Gangnam-gu, Seoul, Republic of Korea), and the resulting nucleotide sequences were compared with reference sequences in the GenBank using the BLASTN tool.

### 2.5. Fungal Culture

A toxigenic isolate of *Aspergillus flavus* A14 (GenBank accession number MW386304), isolated from cashews in our previous research [38], was used in this study. The fungal culture was preserved on potato dextrose agar (PDA; Oxoid Ltd., Basingstoke, Hampshire, UK) medium at 4 °C.

### 2.6. Antagonistic Activity of Weevil Gut Bacteria

The antagonistic activity of the weevil gut bacterial isolates against *A. flavus* was evaluated using a dual culture technique [39]. Briefly, overnight culture of a bacterial isolate was streaked on an NA plate with a diameter of 90 mm, about 10 mm from the outer edge. After that, a 6-mm diameter agar disc taken from a 7-day-old *A. flavus* culture was kept on the opposite side in the same plate, about 10 mm from the Petri plate margin, and incubated at 27 °C for 5 days. Subsequently, the radial growth of the fungus was measured. The NA plate inoculated with *A. flavus* disc alone served as a control. Each treatment was replicated three times. The percentage of growth inhibition was calculated by using the following formula: (C − T)/C × 100, where C and T were the growth of *A. flavus* in the control and in the presence of antagonist, respectively.

### 2.7. Scanning Electron Microscopy (SEM)

The morphological changes in the hyphal structures of *A. flavus* following exposure to the antagonistic weevil gut bacterial isolates were examined using SEM. Mycelial plugs (6 mm) of *A. flavus* were taken from the inhibition zone margin in the dual culture plate and processed according to the method described by Bozzola and Russell [40]. The samples were then examined using a JEOL JSM 4500LV SEM (JEOL Ltd., Peabody, MA, USA) at 20 KV accelerating voltage, and micrographs were obtained. *A. flavus* culture that was cultivated in the absence of bacteria was used as a control.

### 2.8. Testing AFB1-Binding Ability of Bacterial Isolates

Each bacterial isolate was cultured in 10 mL of nutrient broth (NB; Oxoid, UK) at 30 °C on a shaker for 24 h, and the bacterial population was determined using a spectrophotometer at 600 nm. One hundred µL of bacterial culture (OD_600_ of 0.5) was transferred to a 2.0 mL sterile centrifuge tube containing 1 mL of nutrient broth, mixed with 1 µL of AFB1 (1000 ppb; Sigma-Aldrich, St. Louis, MO, USA), and kept at 30 °C in a shaker (170 rpm) for 48 h. After incubation, the tube was centrifuged at 14,000 rpm for 10 min, and the supernatant was collected. Subsequently, an equal volume of chloroform was added to the supernatant, mixed well, and briefly centrifuged. The chloroform fraction was collected in a new tube and dried in a water bath at 60 °C. The residue in the tube was dissolved in methanol, and AFB1 content in the sample was determined by liquid chromatography-mass spectrometry (LC-MS) as per the analysis conditions specified by Al-Mamari et al. [19]. A control sample was prepared using uninoculated NB (1 mL) containing AFB1 (1000 ppb) and processed in the same manner. Each treatment was replicated three times.

### 2.9. Testing AFB1 Degradation by Bacterial Culture Supernatants

Each bacterial isolate was cultured in 10 mL of NB in a 15 mL sterile centrifuge tube at 30 °C for 48 h on a shaker. After incubation, the culture (OD_600_ of 1.0) was centrifuged at 14,000 rpm for 10 min, and cell-free supernatant was collected. To 1 mL of the supernatant, 1 µL of AFB1 (1000 ppb) was added and incubated at 30 °C for 48 h. Subsequently, the AFB1 in the mixture was extracted using chloroform (1:1, *v*/*v*), and its content was determined by LC-MS, and the degraded products of AFB1 were analyzed as described by Al-Mamari et al. [19]. A control sample was prepared using uninoculated NB (1 mL) containing AFB1 (1000 ppb) and processed in the same manner. Each treatment was replicated three times.

### 2.10. LC-MS Analysis of AFB1 Degraded Products

The AFB1 degradation products were analyzed using an Agilent 1290 Infinity II LC system coupled to an Agilent 6460 Triple quadrupole mass spectrometer (Agilent Technologies, Lautengartenstrasse, Basel, Switzerland). A reverse-phase Symmetry C8 5 µm, 3 mm × 150 mm column (Waters, Milford, MA, USA) was used to separate AFB1. The column oven (G1316C) was maintained at 45 °C during the analysis. Mobile phase A consisted of 0.1% formic acid in acetonitrile, while mobile phase B comprised 0.1% formic acid in HPLC grade water, with a solvent flow rate of 0.5 mL/min using a Quaternary pump (G4204A). An autosampler (G4226A) injected 10 µL of each sample. Mass spectrometry was operated in ESI positive ion mode, with the source temperature set at 300 °C and the ion-spray voltage at 4000 V. MassHunter workstation Qualitative analysis ver 6.0.633.0 was used for data acquisition.

### 2.11. Testing Production of Antifungal Volatile Organic Compounds (VOC) by Bacterial Isolates

The production of antifungal VOCs by the bacterial isolates from the weevil gut against *A. flavus* was assessed using the two-sealed base-plates assay [41]. Briefly, 100 μL of overnight bacterial culture (OD_600_ 0.5) was spread onto NA medium using a sterile spreader. A mycelial disc (6 mm diameter) of *A. flavus*, taken from a 7-day-old culture, was placed in the center of a PDA medium. The lids of both inoculated Petri plates were removed, and the base plates were aligned and sealed firmly with two layers of parafilm. The plates were then incubated for 5 days at 27 °C. Subsequently, the diameter of *A. flavus* growth was measured using a ruler, and the inhibition percentage was calculated. The control consisted of a disc of *A. flavus* on a PDA plate covered with an uninoculated NA plate. Each treatment was replicated three times.

### 2.12. Profiling of VOCs of Rice Weevil Gut Bacteria

The VOCs produced by the efficient gut bacterial isolates viz., *B. subtilis* RWGB1, *B. oceanisediminis* RWGB2, and *P. aeruginosa* RWGB4 were analyzed by headspace solid-phase microextraction-gas chromatography-mass spectrometry (HS-SPME-GC-MS). Fifteen ml of sterilized NB in a 40-mL GC-MS vial was inoculated with 100 μL of overnight bacterial culture (OD_600_ 0.5) and incubated at 30 °C in a shaker for 72 h. A vial containing uninoculated NB served as the control. The VOCs released by each bacterium were collected separately, according to Jayakumar et al. [42]. The collected VOCs were then analyzed by GC-MS using a Shimadzu GC-2010 Plus Gas Chromatograph (Shimadzu Corporation, Kyoto, Japan) equipped with a GCMS-QP2010 ULTRA MS and Rtx-5MS capillary column (30 m × 0.25 mm; 0.25 μm), as described by Al-Rashdi et al. [43]. The NIST 2011 v.2.3 and Wiley 9th edition mass spectral libraries were used for compound identification.

### 2.13. Statistical Analysis

Minitab statistical software v21.3 (Minitab Inc., State College, PA, USA) was used for statistical analysis. The data were analyzed through one-way ANOVA, and the differences between treatment means were determined using Tukey’s test (*p* < 0.05).

## 3. Results

### 3.1. Molecular Identification of Rice Weevil and Its Gut Bacterial Isolates

The rice weevil was collected from AFB1-contaminated corn kernels, and its identity was confirmed by mitochondrial cytochrome oxidase I (COI) gene sequence analysis. The nucleotide sequence showed 100% identity with the sequences of *Sitophilus oryzae* (GenBank accession number PP577652). Four bacterial isolates designated RWGB1, RWGB2, RWGB3, and RWGB4, with distinct morphological features, were obtained from the gut of the rice weevil on the NA medium. These bacterial isolates were identified as *Bacillus subtilis* (RWGB1; 100% identity), *Bacillus oceanisediminis* (RWGB2; 99.5% identity), *Bacillus firmus* (RWGB3; 99.8% identity) and *Pseudomonas aeruginosa* (RWGB4; 99.8% identity) based on their 16S rRNA gene sequences, which were submitted to GenBank (accession numbers: OR751661, OR742090, OR742096, and OR742110) (Table 1).

### 3.2. Antagonistic Activity of Bacterial Isolates

**Figure 1:**
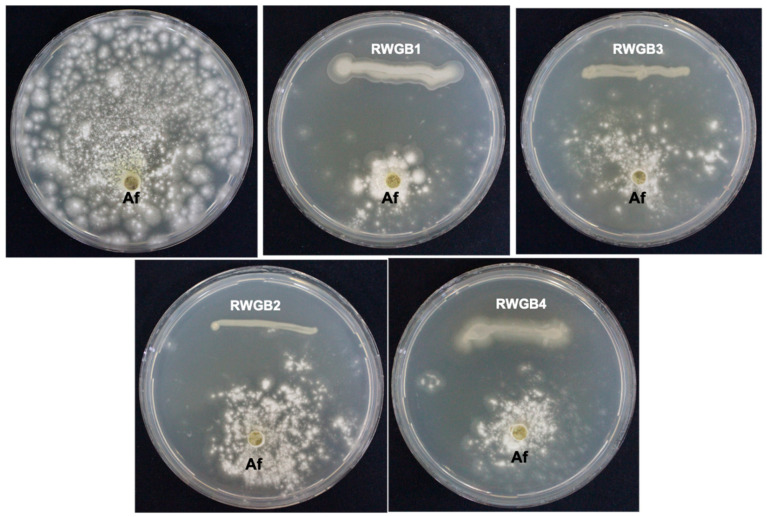
Inhibitory effect of rice weevil gut bacterial isolates on *Aspergillus flavus* (Af).

The ability of the rice weevil gut bacterial isolates to suppress the growth of *A. flavus* was tested in vitro using the dual culture technique. All four bacterial isolates exhibited inhibitory activity against *A. flavus*, resulting in clear inhibition zones. Among them, *P. aeruginosa* RWGB4 showed the highest level of inhibition of *A. flavus* growth (75%), followed by *B. subtilis* RWGB1(71.9%), *B. firmus* RWGB3 (70.3%) and *B. oceanisediminis* RWGB2 (57.8%), compared to the control (Table 2; Figure 1).

**Figure 2:**
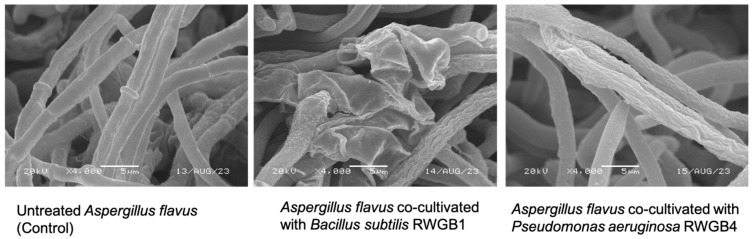
Scanning electron micrographs displaying abnormalities in *Aspergillus flavus* hyphae following co-cultivation with bacterial isolates from the rice weevil gut.

SEM analysis of *A. flavus* hyphae at the inhibition zone in the dual culture plate revealed that all the bacterial isolates induced morphological changes in the hyphae, characterized by wrinkling of the surface, shriveling, twisting, and disintegration compared to the smooth and normal hyphae in the control. The scanning electron micrographs of *A. flavus* hyphae co-cultivated with *B. subtilis* RWGB1 and *P. aeruginosa* RWGB4 and the untreated control are shown in Figure 2.

### 3.3. Removal of AFB1 by the Rice Weevil Gut Bacterial Isolates

The ability of the bacterial isolates from the gut of the rice weevil to remove AFB1 from the culture medium was assessed in vitro. The results showed that all four bacterial isolates could significantly reduce the level of AFB1. Among them, *B. subtilis* RWBG1 exhibited the highest efficiency, removing 84.2% of AFB1. *B. oceanisediminis* RWGB2, *B. firmus* RWGB3, and *P. aeruginosa* RWGB4 removed 66.5%, 63.6%, and 48.9% of AFB1, respectively (Table 3).

### 3.4. Degradation of AFB1 by Culture Supernatants of the Rice Weevil Gut Bacteria

The supernatant of *P. aeruginosa* (RWBG4) culture exhibited the highest level of AFB1-degrading ability, with 87.1% degradation, followed by *B. firmus* RWGB3 (43.5%) and *B. oceanisediminis* RWGB2 (23.2%) (Table 4). The supernatant of *B. subtilis* RWGB1 culture was the least effective, with 8.4% degradation.

### 3.5. LC/MS Analysis of AFB1 Degraded Products

**Figure 3:**
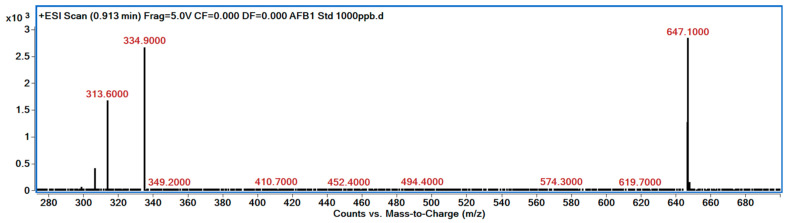
Mass spectrum of AFB1 treated with uninoculated nutrient broth (control) showing AFB1 protonated molecular ion (M + H) with *m*/*z* 313.6, AFB1 sodium adduct ion (M + Na) with *m*/*z* 334.9, and AFB1 dimer with sodium adduct (2M + Na) with *m*/*z* 647.1.

**Figure 4:**
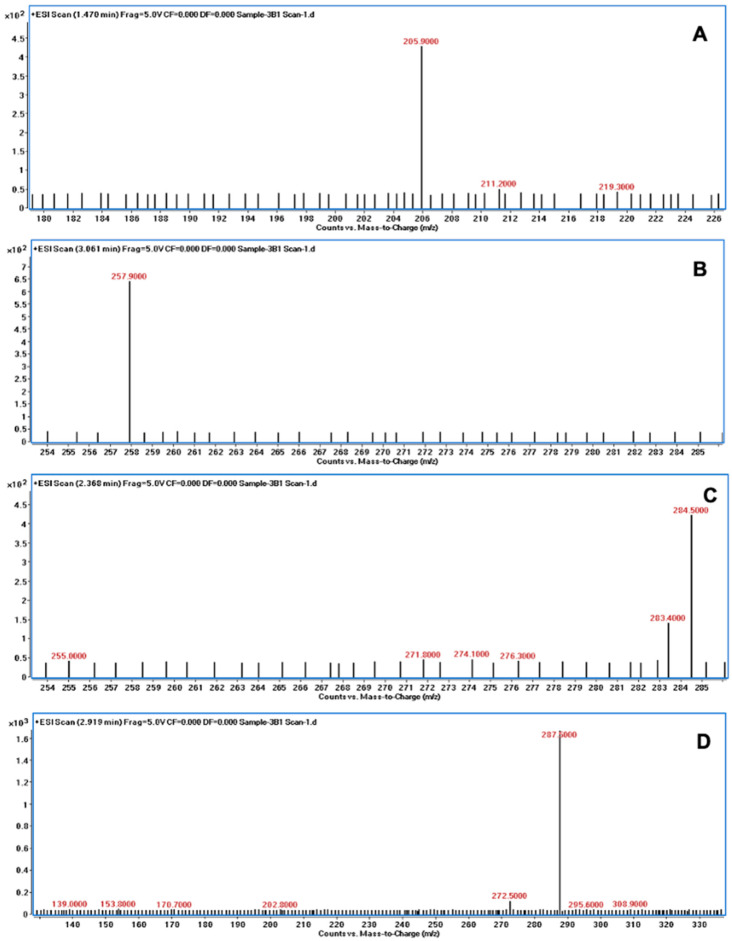
Mass spectra of AFB1 treated with the culture supernatant of *Bacillus subtilis* RWGB1 showing AFB1 degradation products with *m*/*z* 205.9 (**A**), *m*/*z* 257.9 (**B**), *m*/*z* 284.5 (**C**), and *m*/*z* 287.5 (**D**).

**Figure 5:**
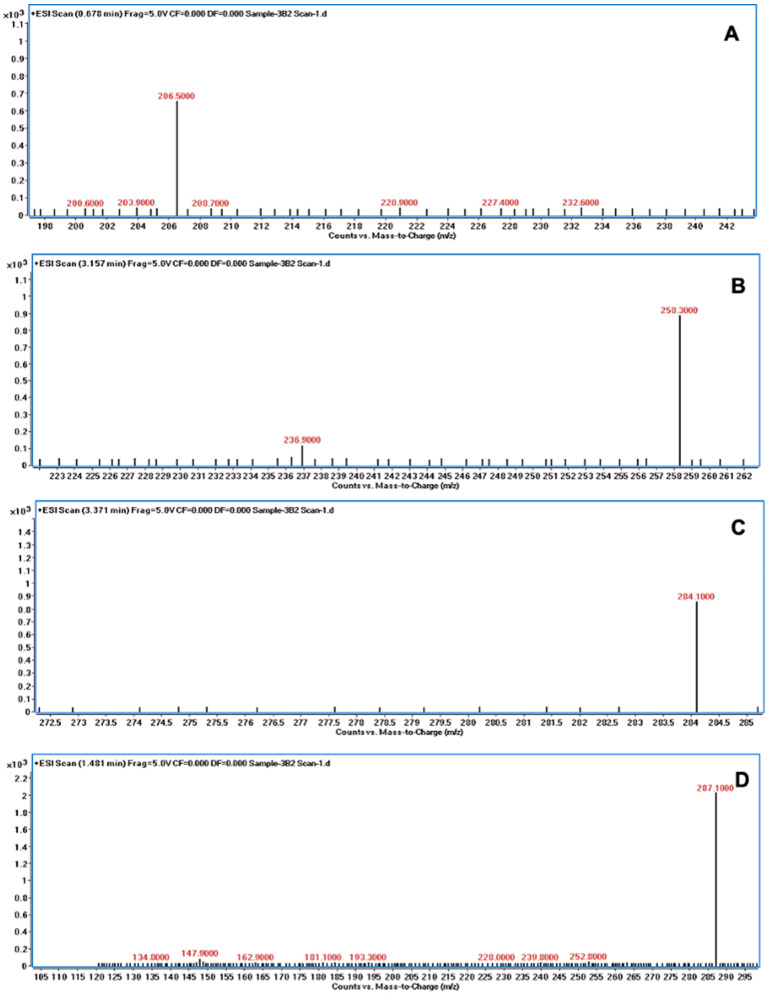
Mass spectra of AFB1 treated with the culture supernatant of *Bacillus oceanisediminis* RWGB2 showing AFB1 degradation products with *m*/*z* 206.5 (**A**), *m*/*z* 258.3 (**B**), *m*/*z* 284.1 (**C**), and *m*/*z* 287.1 (**D**).

**Figure 6:**
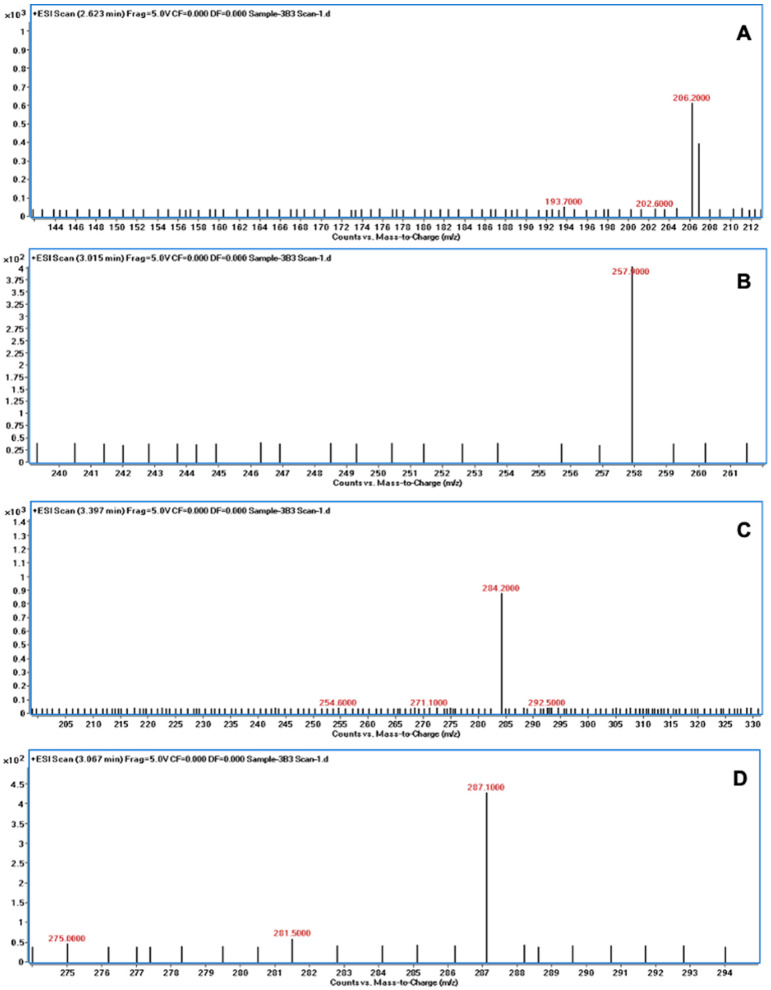
Mass spectra of AFB1 treated with the culture supernatant of *Bacillus firmus* RWGB3 showing AFB1 degradation products with *m*/*z* 206.2 (**A**), *m*/*z* 257.9 (**B**), *m*/*z* 284.2 (**C**), and *m*/*z* 287.1 (**D**).

**Figure 7:**
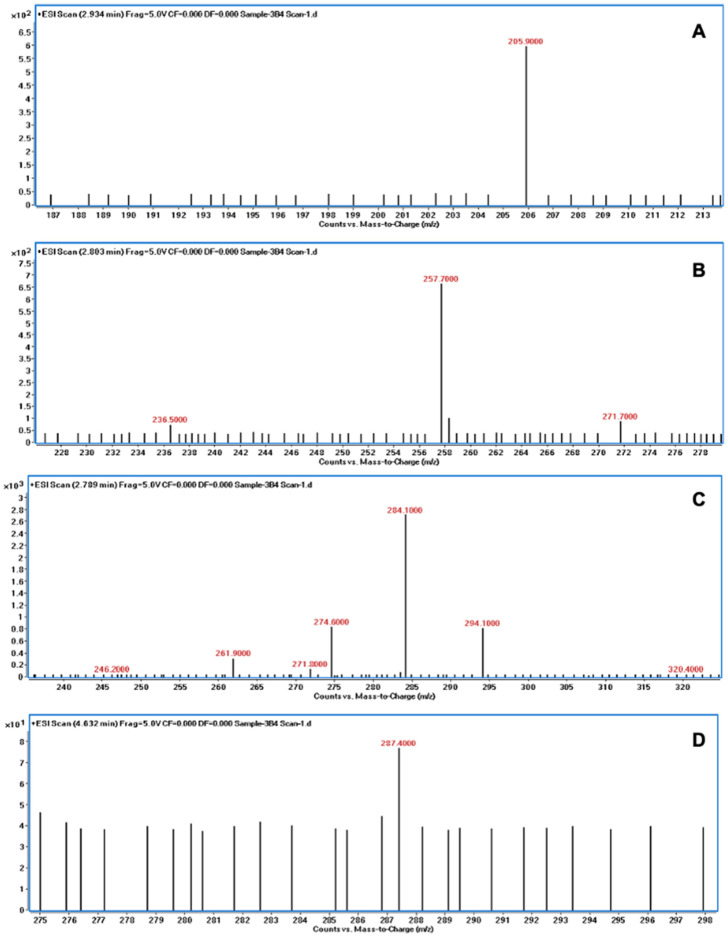
Mass spectra of AFB1 treated with the culture supernatant of *Pseudomonas aeruginosa* RWGB4 showing AFB1 degradation products with *m*/*z* 205.9 (**A**), *m*/*z* 257.7 (**B**), *m*/*z* 284.1 (**C**), and *m*/*z* 287.4 (**D**).

The degradation of AFB1 was verified using LC/MS after incubating AFB1 with the bacterial culture supernatants. The mass spectrum of AFB1 treated with uninoculated nutrient broth (control) displayed the aflatoxin protonated molecular ion (M + H) with *m*/*z* 313.6, AFB1 sodium adduct ion (M + Na) with *m*/*z* 334.9, and AFB1 dimer with sodium adduct (2M + Na) with *m*/*z* 647.1 (Figure 3). In contrast, the degraded products of AFB1, including *m*/*z* 206, 258, 284, and 287, were detected following treatment with the culture supernatants of *B. subtilis* RWGB1, *B. oceanisediminis* RWGB2, *B. firmus* RWGB3, and *P. aeruginosa* RWGB4, compared to the control, confirming the degradation of AFB1 (Figure 4, Figure 5, Figure 6 and Figure 7).

### 3.6. Production of Antifungal VOCs by the Rice Weevil Gut Bacteria

The production of antifungal VOCs by the bacterial isolates from the rice weevil gut bacteria against *A. flavus* was evaluated using the two-sealed base-plates assay. The results showed that *B. subtilis* RWGB1, *B. oceanisediminis* RWGB2, and *P. aeruginosa* RWGB4 emitted antifungal VOCs that suppressed the growth of *A. flavus*, resulting in an inhibition of 78–82% (Table 5). However, the VOCs released by *B. firmus* RWGB3 were not effective against *A. flavus*.

### 3.7. HS-SPME-GC-MS Analysis of VOCs

**Figure 8:**
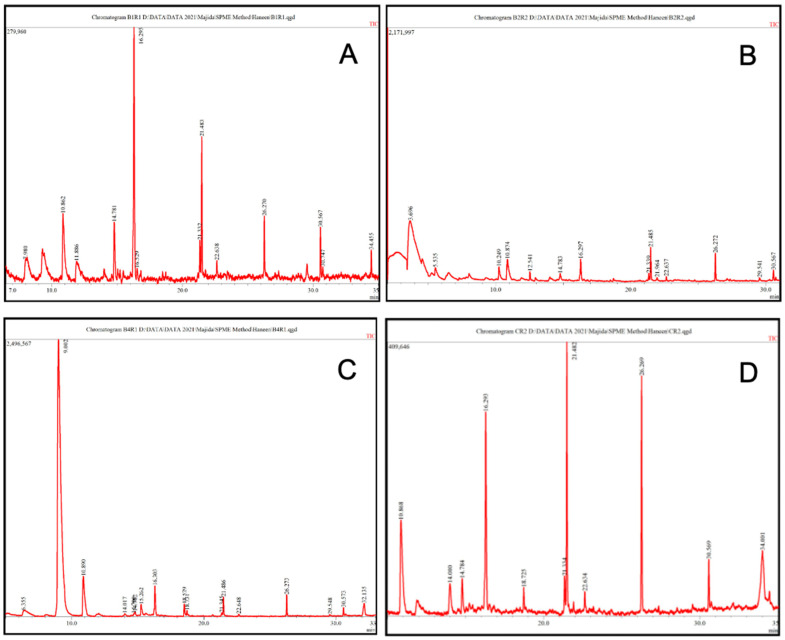
Gas chromatography-mass spectrometry total ion chromatograms of VOCs detected in the headspace of *Bacillus subtilis* RWGB1 (**A**), *Bacillus oceanisediminis* RWGB2 (**B**), *Pseudomonas aeruginosa* RWGB4 (**C**), and control (**D**).

Analysis of the VOCs released by the effective bacterial isolates from the rice weevil gut using HS-SPME-GC-MS revealed that silane, trimethyl(1-methyl-1-propenyl)-, (E)- was the major compound (33.43%) produced by *B. subtilis* RWBG1, followed by silane, tetramethyl- (13.62%) and pentamethyldisilane (13.34%) (Figure 8A; Table 6). *B. oceanisediminis* RWGB2 predominantly produced butanoic acid, 2-methyl-, ethyl ester (69.98%) (Figure 8B; Table 7). *P. aeruginosa* RWBG4 was found to primarily produce 1-decanol (81.77%) (Figure 8C; Table 8). Silane, trimethyl(1-methyl-1-propenyl)-, (E)- (18.76%), silane, tetramethyl- (17.97%), pentamethyldisilane (17.75%) and hexane, 1-chloro-5-methyl- (12.83%) were identified as the major compounds in the uninoculated nutrient broth (control) (Figure 8D; Table 9).

## 4. Discussion

Decontamination of aflatoxins from food products presents a challenge due to their high stability under normal food processing conditions. Biological degradation of aflatoxins, using microorganisms or their byproducts, is considered an ideal method to reduce the toxic effects of aflatoxins due to their effectiveness and environmental friendliness [13,18,44,45]. In this study, four bacterial species, viz., *B. subtilis* RWGB1, *B. oceanisediminis* RWGB2, *B. firmus* RWGB3, and *P. aeruginosa* RWGB4, residing in the gut of the rice weevil were isolated. These gut-inhabiting bacterial isolates exhibited direct antagonistic activity against *A. flavus*, as evidenced by the production of inhibition zones in the in vitro co-culture assay. The antagonistic activity of *B. subtilis* [46], *B. oceanisediminis* [47], *B. firmus* [48], and *P. aeruginosa* [49], isolated from different sources, against a diverse range of fungi has been described. Several studies have reported that symbiotic bacteria associated with the gut of insects exhibit antagonistic activity against fungi [50,51,52]. For example, Huang et al. [50] demonstrated that *B. subtilis* from the gut of *Blattella germanica* retarded the growth of *Beauveria bassiana*. Miller et al. [53], while examining the antifungal activity of honey bee-associated bacteria, observed that the bacterial symbiont *Bombella apis* could suppress the growth of *A. flavus* and *Beauveria bassiana* in vitro. Amer et al. [51] showed that *Klebsiella pneumonia* isolated from the *Periplaneta americana* gut exhibited inhibitory activity against *A. flavus*. Shehabeldine et al. [52] found that cell-free culture supernatants of a few bacterial species, including *Pseudomonas aeruginosa* obtained from the gut of *Apis mellifera* displayed in vitro antifungal activity against *Aspergillus* sp., the causal agent of Stonebrood disease of honey bee. The inhibitory activity of the rice weevil gut-associated bacterial isolates against *A. flavus* in the dual culture assay in this study is likely due to their production of diffusible antifungal metabolites [54] and depletion of essential nutrients in the media [55]. The differences in *A. flavus* growth inhibition could be attributed to differences in the concentration and toxicity of the metabolites produced by these bacterial isolates.

SEM analysis of *A. flavus* hyphae sampled from the inhibition zone in a dual culture assay plate showed aberrant morphologies, including wrinkling of the surface, shrinkage, and distortion. Similar findings were reported by Al-Daghari et al. [56] when *P. aphanidermatum*, the damping-off pathogen of cucumber, was co-cultivated with antagonistic strains of *Serratia marcescens* and *Pseudomonas* spp. Likewise, shrinkage of *P. aphanidermatum* mycelium was observed when the oomycete was co-cultured with an antagonistic fungus, *Aspergillus terreus* [57]. The shrinkage of fungal hyphae may be attributed to the loss of internal cell contents [58].

Several microorganisms with the potential of degrading AFB1 have been isolated from various sources, including soil [59,60], fish gut [61], and fermented foods [19]. These microorganisms include *Saccharomyces cerevisiae* [62], lactic acid bacteria [63,64,65], *B. subtilis* [19,61,66], *B. licheniformis* [17], *B. amyloliquefaciens* [67], *P. putida* [59], and *Streptomyces* spp. [68]. The bacterial isolates from the rice weevil gut in this study also demonstrated the ability to reduce AFB1 levels. *B. subtilis* RWBG1 was highly efficient, removing 84.2% of AFB1. *B. oceanisediminis* RWGB2, *B. firmus* RWGB3, and *P. aeruginosa* RWGB4 removed 66.5%, 63.6%, and 48.9% of AFB1, respectively. These results are consistent with the previous reports that indicated the degradation of AFB1 by various bacterial strains [17,19,60,61,69,70]. El-Nezami et al. [69] reported that *Lactobacillus rhamnosus* GG, a lactic acid bacterium, removed 80% of AFB1 from the toxin-amended culture medium. Gao et al. [61] found that *B. subtilis* from fish gut degraded 81.5% of AFB1. Rao et al. [17] demonstrated that *B. licheniformis* degraded over 90% of AFB1. Ali et al. [60] reported that *P. fluorescens* strain SZ1 isolated from soil degraded 100% of AFG1 and 99% of aflatoxin B1, B2, and G2. Al-Mamari et al. [19] found that live cells of *B. subtilis* YGT1 obtained from yogurt degraded 83.8% of AFB1 after 48 h of incubation at 30 °C. El-Nezami et al. [71] showed that even heat- and acid-treated dead cells of *L. rhamnosus* GG could bind AFB1. The peptidoglycan in the cell wall of *L. rhamnosus* GG has been reported as crucial for binding AFB1 [72].

The results of the present study also showed that the culture supernatant of *P. aeruginosa* RWBG4 exhibited the highest AFB1-degrading capability (87.1%), followed by *B. firmus* RWGB3 (43.5%) and *B. oceanisediminis* RWGB2 (23.2%). *B. subtilis* RWGB1 culture supernatant was the least effective, with 8.4% degradation. Xu et al. [73] showed that *B. shackletonii* L7 significantly reduced AFB1 levels (92.1%), with the culture supernatant degrading more AFB1 than live cells or cell extracts. Al-Mamari et al. [19] reported that the culture supernatant of *B. subtilis* YGT1 degraded 81.3% of AFB1 in culture medium after 48 h of incubation at 30 °C. The furofuran and lactone rings are the critical sites for the toxic effects of aflatoxins. Modifications to these ring structures typically lead to the loss of the toxic effects of aflatoxins [74,75]. Several bacteria and fungi have been reported to degrade aflatoxins by modifying the lactone ring or cyclopentanone ring structures [17,70,72,76]. The results of the present study suggest that *B. subtilis* RWGB1 eliminates AFB1 primarily through physical binding, while *P. aeruginosa* RWBG4, *B. firmus* RWGB3, and *B. oceanisediminis* RWGB2 degrade it through physical binding and by using their extracellular metabolites.

The degradation of AFB1 upon incubation with the bacterial culture supernatants was confirmed by LC/MS analysis. AFB1 degraded products *m*/*z* 206, 258, 284, and 287 were detected following treatment with the culture supernatants of the rice weevil gut bacteria, compared to AFB1-treated with uninoculated nutrient broth (control), confirming the degradation of AFB1. Several studies documented the molecular structures of AFB1 degradation products [77,78,79,80]. Cucullu et al. [78] showed that ammoniation treatment resulted in the formation of dihydro-4-hydroxy-6-methoxyfuro [2,3-b] benzofuran (MW 206) from AFB1. Lee et al. [77] documented the generation of a product with a molecular mass of 286 from AFB1 following treatment with NH_4_OH. Wang et al. [81] reported that live cells and the culture supernatant of *Escherichia coli* strain CG1061 isolated from chicken cecum showed 93.7% and 61.8% degradation of AFB1, respectively. AFB1 was bio-transformed into AFD1 and other metabolites such as C_17_H_12_O_7_, C_26_H_25_N_3_O_12_S, C_17_H_15_O_5_, and C_8_H_4_O_3_. AFD1 (C_16_H_14_O_5_; *m*/*z* 287.09) is produced by the opening of the lactone ring of AFB1. Al-Owaisi et al. [82], while studying the AFB1-detoxifying potential of herbal medicinal products, proposed a fragmentation pathway of AFB1 which showed the formation of product ions *m*/*z* 284 and *m*/*z* 258 due to two sequential losses of carbon monoxide, followed by the loss of the methoxy moiety (*m*/*z* 228), and a carbon monoxide and a formaldehyde moiety (*m*/*z* 188). The appearance of the product ions with *m*/*z* 206, 258, 284, and 287 following treatment of AFB1 with the culture supernatants of the rice weevil gut bacterial isolates in this study suggests degradation of AFB1.

The in vitro two-sealed base-plates assay in this study showed that *B. subtilis* RWGB1, *B. oceanisediminis* RWGB2, and *P. aeruginosa* RWGB4 produced antifungal volatile compounds that suppressed the growth of *A. flavus*. In contrast, the volatiles from *B. firmus* RWGB3 were not effective against *A. flavus.* The production of VOCs is recognized as an important mechanism by which antagonistic bacterial isolates suppress plant pathogenic fungi [41,42,56,83,84,85,86]. These VOCs are gaseous, lipophilic, low-molecular-weight (<300 Da) compounds [87] that disrupt fungal cell membranes, leading to the leakage of cell contents and, eventually, cell death [88,89]. Strobel et al. [90] reported the synergistic antimicrobial activity of VOCs against plant pathogens.

Profiling of the VOCs produced by the rice weevil gut bacterial isolates in this study revealed that *B. subtilis* RWBG1 primarily produced silane, trimethyl(1-methyl-1-propenyl)-, (E)-, silane, tetramethyl-, and pentamethyldisilane. *B. oceanisediminis* RWGB2 predominantly produced butanoic acid, 2-methyl-, ethyl ester. *P. aeruginosa* RWBG4 was found to produce 1-decanol as its major component. Antimicrobial activity of butanoic acid, 2-methyl-, ethyl ester (C_7_H_14_O_2_; MW 130.1849; syn: Butyric acid, 2-methyl-, ethyl ester) has been reported [90,91]. Li et al. [92] reported the antifungal effect of 1-decanol (C_10_H_22_O; MW 158.2811; syn: Decyl alcohol; Capric alcohol; Decanol) from antagonistic bacterium *Bacillus velezensis* against *Verticillium dahliae* and *Fusarium oxysporum*. Gao et al. [93] reported that the major VOCs produced by *B. subtilis* strain CF-3 that showed antagonistic activity against *Monilinia fructicola* and *Colletotrichum gloeosporioides* were (*S*)-1-octanol, benzoic acid, 2,4-di-*tert*-butylphenol, benzaldehyde, and benzothiazole. However, in this study, silane, trimethyl(1-methyl-1-propenyl)-, (E)-, silane, tetramethyl-, and pentamethyldisilane were identified as major compounds produced by *B. subtilis* RWBG1. Similarly, Al-Daghari et al. [56] reported that an antagonistic strain of *P. aeruginosa* B1-SQU obtained from cabbage rhizosphere, which showed inhibitory activity against *P. aphanidermatum*, produced 1-Butanol, 3-methyl- (syn: Isopentanol/Isoamyl alcohol) and dimethyl disulfide as the major compounds. Al-Rashdi et al. [86] showed that *P. aeruginosa* PC5 obtained from *Prosopis cineraria* produced dimethyl disulfide as the major VOC, followed by n-hexadecanoic acid and di-tert-butyl decarbonate, whereas 1-decanol was identified as the major component in this study. The variations in the chemical composition of these VOCs might be due to the difference in the type of culture medium, as well as the duration of incubation [87]. In this study, silane, trimethyl(1-methyl-1-propenyl)-, (E)-, silane, tetramethyl-, and pentamethyldisilane were found as major components in both *B. subtilis* RWBG1 and the control. However, VOCs from *B. subtilis* RWBG1 contained an antifungal compound, decane, 2,3,5,8-tetramethyl-(C_14_H_30_; MW 198.3880; syn: 2,3,5,8-Tetramethyl-decane) in addition to the above compounds. Sholkamy et al. [94] reported that volatile metabolites from *Streptomyces* sp. nkm1 inhibited the growth of plant pathogenic fungi, including *A. flavus*, and the bioactive fraction contained decane, 2,3,5,8-tetramethyl- as one of its components.

## 5. Conclusions

In this study, *B. subtilis* RWBG1, *B. oceanisediminis* RWBG2, *B. firmus* RWBG3, and *P. aeruginosa* RWBG4, isolated from the gut of the rice weevil, effectively suppressed the growth of *A. flavus* in vitro. In addition, both live cells and/or the culture supernatant of these bacterial isolates could degrade AFB1 under laboratory conditions. These gut-inhabiting bacterial isolates show promise as potential candidates for AFB1 removal/detoxification. Further studies are needed to examine the mode of action of culture supernatants of the rice weevil gut bacteria on AFB1 and the biological toxicity of the AFB1 products. While *P. aeruginosa* is an opportunistic pathogen known to cause severe acute and chronic infections in humans, several strains of *Bacillus* sp. are considered “generally recognized as safe” (GRAS) organisms. Further exploration of the application of these *Bacillus* spp. isolates in food and feed industries could contribute to safer food and feed production. This appears to be the first report of AFB1 degradation by gut bacteria from the rice weevil.

## Figures and Tables

**Table 1:** Bacterial isolates obtained from the rice weevil gut and identified based on 16S rRNA gene sequences.

Bacterial Isolate	Homologous Microorganism	% Identity	GenBank Accession Number
RWGB1	*Bacillus subtilis*	100%	OR751661
RWGB2	*Bacillus oceanisediminis*	99.5%	OR742090
RWGB3	*Bacillus firmus*	99.8%	OR742096
RWGB4	*Pseudomonas aeruginosa*	99.8%	OR742110

**Table 2:** In vitro antagonistic activity of rice weevil gut bacterial isolates against *Aspergillus flavus*.

Treatment	Radial Growth of *A. flavus* (cm)	% Inhibition
*Bacillus subtilis* RWGB1	1.8 b	71.9
*Bacillus oceanisediminis* RWGB2	2.7 b	57.8
*Bacillus firmus* RWGB3	1.9 b	70.3
*Pseudomonas aeruginosa* RWGB4	1.6 b	75.0
Control	6.4 a	-

Values with the same letter are not significantly different from each other at *p* < 0.05 by Tukey’s test.

**Table 3:** Removal of aflatoxin B1 by live bacterial cells from the rice weevil gut.

Bacterial Isolate	AFB1 Recovered (ppb)	% Reduction
*Bacillus subtilis* RWGB1	154.7 e	84.2
*Bacillus oceanisediminis* RWGB2	328.1 d	66.5
*Bacillus firmus* RWGB3	355.7 c	63.6
*Pseudomonas aeruginosa* RWGB4	500.2 b	48.9
Control	978.1 a	-

Values with the same letter are not significantly different from each other at *p* < 0.05 by Tukey’s test.

**Table 4:** Degradation of aflatoxin B1 by cell-free culture supernatants of bacterial isolates from the rice weevil gut.

Bacterial Isolate	AFB1 Recovered (ppb)	% Degradation
*Bacillus subtilis* RWGB1	895.8 b	8.4
*Bacillus oceanisediminis* RWGB2	750.8 c	23.2
*Bacillus firmus* RWGB3	552.4 d	43.5
*Pseudomonas aeruginosa* RWGB4	126.2 e	87.1
Control	978.1 a	-

Values with the same letter are not significantly different from each other at *p* < 0.05 by Tukey’s test.

**Table 5:** Production of antifungal VOCs by the rice weevil gut bacteria against *Aspergillus flavus*.

Treatments	Diameter Growth of *A. flavus* (cm)	% Reduction
*Bacillus subtilis* RWGB1	1.6 b	81
*Bacillus oceanisediminis* RWGB2	1.8 b	78.6
*Bacillus firmus* RWGB3	8.4 a	0
*Pseudomonas aeruginosa* RWGB4	1.5 b	82.1
Control	8.4 a	-

Values with the same letter are not significantly different from each other at *p* < 0.05 by Tukey’s test.

**Table 6:** VOCs detected in the headspace of *Bacillus subtilis* RWGB1.

S. No.	Compound	Retention Time (min)	Peak Area %
1	Decane, 2,3,5,8-tetramethyl-	7.98	4.84
2	Silane, tetramethyl-	10.862	13.62
3	3-Phenyl-2-cyclopenten-1-one	11.886	4.81
4	Dodecane, 4,6-dimethyl-	14.781	8.63
5	Silane, trimethyl(1-methyl-1-propenyl)-, (E)-	16.295	33.43
6	Dodecane, 2,7,10-trimethyl-	16.529	1.32
7	Nonane, 3-methyl-5-propyl-	21.332	4.12
8	Pentamethyldisilane	21.483	13.34
9	Heptadecane, 2,6,10,15-tetramethyl-	22.638	1.37
10	Disilane, pentamethyl-	26.27	5.37
11	2-Methoxy-1,3-dioxolane	30.567	4.9
12	4,6-Decadiyne	30.747	1.63
13	1-Deoxyhexitol	34.455	2.62

**Table 7:** VOCs detected in the headspace of *Bacillus oceanisediminis* RWGB2.

S. No.	Compound	Retention Time (min)	Peak Area %
1	Butanoic acid, 2-methyl-, ethyl ester	3.696	69.98
2	Hexanal	5.535	2.43
3	Nonanal	10.249	2.92
4	Silane, tetramethyl-	10.874	7.53
5	N-Methyl-dl-leucine	12.541	1.02
6	Dodecane, 4,6-dimethyl-	14.783	1.25
7	Silane, trimethyl(1-methyl-1-propenyl)-, (E)-	16.297	4.25
8	Nonane, 3-methyl-5-propyl-	21.339	0.78
9	Pentamethyldisilane	21.485	3.96
10	Cycloheptanol	21.964	0.23
11	Sulfurous acid, 2-ethylhexyl undecyl ester	22.637	0.41
12	Disilane, pentamethyl-	26.272	3.43
13	1-Bromo-1-phenylpropane	29.541	0.45
14	Methoxymethyl trimethylsilane	30.567	1.36

**Table 8:** VOCs detected in the headspace of *Pseudomonas aeruginosa* RWGB4.

S. No.	Compound	Retention Time (min)	Peak Area %
1	1,4-Dioxan-2-ol, TMS derivative	6.355	0.17
2	1-Decanol	9.002	81.77
3	Silane, tetramethyl-	10.89	7.16
4	1-Deoxyhexitol	14.017	0.22
5	1,9-Nonanediol	14.7	0.13
6	Dodecane, 4,6-dimethyl-	14.782	0.38
7	2-Tridecanone	15.262	1.15
8	Silane, trimethyl(1-methyl-1-propenyl)-, (E)-	16.303	2.66
9	Formic acid, neryl ester	18.529	1.04
10	1,3-Dioxolane, 2-pentadecyl-	18.731	0.32
11	Nonane, 3-methyl-5-propyl-	21.345	0.16
12	Pentamethyldisilane	21.486	1.24
13	Sulfurous acid, 2-ethylhexyl undecyl ester	22.648	0.12
14	Disilane, pentamethyl-	26.273	1.26
15	1-Bromo-1-phenylpropane	29.548	0.06
16	Methoxymethyl trimethylsilane	30.573	0.49
17	2-Methoxy-1,3-dioxolane	32.135	1.67

**Table 9:** VOCs detected in the headspace of uninoculated nutrient broth (control).

S. No.	Compound	Retention Time (min)	Peak Area %
1	Silane, tetramethyl-	10.868	17.97
2	Trimethylsilyl 2-(2-butoxyethoxy)acetate	14	4.88
3	Dodecane, 4,6-dimethyl-	14.784	3.75
4	Silane, trimethyl(1-methyl-1-propenyl)-, (E)-	16.293	18.76
5	1,3-Dioxolane, 2-pentadecyl-	18.725	1.54
6	Nonane, 3-methyl-5-propyl-	21.334	2.69
7	Pentamethyldisilane	21.482	17.75
8	2,3,5,8-Tetramethyldecane	22.634	1.2
9	Unidentified	26.269	15.31
10	2-Methoxy-1,3-dioxolane	30.569	3.32
11	Hexane, 1-chloro-5-methyl-	34.001	12.83

## Data Availability

Data are contained within the article.

## References

[1] Luo S., Du H., Kebede H., Liu Y., Xing F. (2021). Contamination status of major mycotoxins in agricultural product and food stuff in Europe. Food Control.

[2] Abrehame S., Manoj V.R., Hailu M., Chen Y.Y., Lin Y.C., Chen Y.P. (2023). Aflatoxins: Source, detection, clinical features and prevention. Processes.

[3] Benkerroum N. (2020). Chronic and acute toxicities of aflatoxins: Mechanisms of action. Int. J. Environ. Res. Public Health.

[4] Marr K.A., Platt A., Tornheim J.A., Zhang S.X., Datta K., Cardozo C., Garcia-Vidal C. (2021). Aspergillosis complicating severe coronavirus disease. Emerg. Infect. Dis..

[5] Pickova D., Ostry V., Malir F. (2021). A recent overview of producers and important dietary sources of aflatoxins. Toxins.

[6] Shabeer S., Asad S., Jamal A., Ali A. (2022). Aflatoxin contamination, its impact and management strategies: An updated review. Toxins.

[7] Chanda A., Roze L.V., Linz J.E. (2010). A possible role for exocytosis in aflatoxin export in *Aspergillus parasiticus*. Eukaryot. Cell.

[8] Kumar A., Pathak H., Bhadauria S., Sudan J. (2021). Aflatoxin contamination in food crops: Causes, detection, and management: A review. Food Prod. Process. Nutr..

[9] Popescu R.G., Rădulescu A.L., Georgescu S.E., Dinischiotu A. (2022). Aflatoxins in feed: Types, metabolism, health consequences in swine and mitigation strategies. Toxins.

[10] Abdin M.Z., Ahmad M.M., Javed S. (2010). Advances in molecular detection of Aspergillus: An update. Arch. Microbiol..

[11] International Agency for Research on Cancer (IARC) (2002). Summaries and Evaluations: Aflatoxins.

[12] Sadeghi E., Solaimanimehr S., Mirzazadeh M., Jamshidpoor S. (2023). The effect of gamma irradiation, microwaves, and roasting on aflatoxin levels in pistachio kernels. World Mycotoxin J..

[13] Song C., Yang J., Wang Y., Ding G., Guo L., Qin J. (2024). Mechanisms and transformed products of aflatoxin B1 degradation under multiple treatments: A review. Crit. Rev. Food Sci. Nutr..

[14] Wu Q., Jezkova A., Yuan Z., Pavlikova L., Dohnal V., Kuca K. (2009). Biological degradation of aflatoxins. Drug Metab. Rev..

[15] Guan S., Zhou T., Yin Y., Xie M., Ruan Z., Young J. (2011). Microbial strategies to control aflatoxins in food and feed. World Mycotoxin J..

[16] Velazhahan R. (2017). Bioprospecting of medicinal plants for detoxification of aflatoxins. Int. J. Nutr. Pharmacol. Neurol. Dis..

[17] Rao K.R., Vipin A.V., Hariprasad P., Appaiah K.A.A., Venkateswaran G. (2017). Biological detoxification of Aflatoxin B1 by *Bacillus licheniformis* CFR1. Food Control.

[18] Guan Y., Chen J., Nepovimova E., Long M., Wu W., Kuca K. (2021). Aflatoxin detoxification using microorganisms and enzymes. Toxins.

[19] Al-Mamari A., Al-Sadi A.M., Al-Harrasi M.M.A., Sathish Babu S.P., Al-Mahmooli I.H., Velazhahan R. (2023). Biodegradation of aflatoxin B1 by *Bacillus subtilis* YGT1 isolated from yoghurt. Int. Food Res. J..

[20] Guerre P. (2020). Mycotoxin and gut microbiota interactions. Toxins.

[21] Guan S., He J., Young J.C., Zhu H., Li X.Z., Ji C., Zhou T. (2009). Transformation of trichothecene mycotoxins by microorganisms from fish digesta. Aquaculture.

[22] Yu H., Zhou T., Gong J., Young C., Su X., Li X.Z., Zhu H., Tsao R., Yang R. (2010). Isolation of deoxynivalenol-transforming bacteria from the chicken intestines using the approach of PCR-DGGE guided microbial selection. BMC Microbiol..

[23] Gao X., Mu P., Wen J., Sun Y., Chen Q., Deng Y. (2018). Detoxification of trichothecene mycotoxins by a novel bacterium, *Eggerthella* sp. DII-9. Food Chem. Toxicol..

[24] Alberts J.F., Engelbrecht Y., Steyn P.S., Holzapfel W.H., Van Zyl W.H. (2006). Biological degradation of aflatoxin B1 by *Rhodococcus erythropolis* cultures. Int. J. Food Microbiol..

[25] El-Deeb B., Altalhi A., Khiralla G., Hassan S., Gherbawy Y. (2013). Isolation and characterization of endophytic *Bacilli* bacterium from maize grains able to detoxify aflatoxin B1. Food Biotechnol..

[26] Samuel M.S., Sivaramakrishna A., Mehta A. (2014). Degradation and detoxification of aflatoxin B1 by *Pseudomonas putida*. Int. Biodeterior. Biodegradation.

[27] Juri F.M.G., Dalcero A.M., Magnoli C.E. (2015). In vitro aflatoxin B1 binding capacity by two *Enterococcus faecium* strains isolated from healthy dog faeces. J. Appl. Microbiol..

[28] Camenzuli L., Van Dam R., de Rijk T., Andriessen R., van Schelt J., van der Fels-Klerx H.J. (2018). Tolerance and excretion of the mycotoxins aflatoxin B1, zearalenone, deoxynivalenol, and ochratoxin A by *Alphitobius diaperinus* and *Hermetia illucens* from contaminated substrates. Toxins.

[29] Evans N.M., Shao S. (2022). Mycotoxin metabolism by edible insects. Toxins.

[30] Engel P., Moran N.A. (2013). The gut microbiota of insects–diversity in structure and function. FEMS Microbiol. Rev..

[31] Siddiqui J.A., Khan M.M., Bamisile B.S., Hafeez M., Qasim M., Rasheed M.T., Rasheed M.A., Ahmad S., Shahid M.I., Xu Y. (2022). Role of insect gut microbiota in pesticide degradation: A review. Front. Microbiol..

[32] Yun J.H., Roh S.W., Whon T.W., Jung M.J., Kim M.S., Park D.S., Yoon C., Nam Y.D., Kim Y.J., Choi J.H. (2014). Insect gut bacterial diversity determined by environmental habitat, diet, developmental stage, and phylogeny of host. Appl. Environ. Microbiol..

[33] Suganthi M., Abirami G., Jayanthi M., Kumar K.A., Karuppanan K., Palanisamy S. (2023). A method for DNA extraction and molecular identification of Aphids. MethodsX.

[34] Folmer O., Black M., Hoeh W., Lutz R., Vrijenhoek R. (1994). DNA primers for amplification of mitochondrial cytochrome c oxidase subunit I from diverse metazoan invertebrates. Mol. Mar. Biol. Biotechnol..

[35] Abbasi I., Halaseh L., Darwish H.M., Matouk I. (2021). Strategy for DNA extraction and detection from insect pests in stored home grain samples. Al-Quds J. Acad. Res..

[36] Frank J.A., Reich C.I., Sharma S., Weisbaum J.S., Wilson B.A., Olsen G.J. (2008). Critical evaluation of two primers commonly used for amplification of bacterial 16S rRNA genes. Appl. Environ. Microbiol..

[37] Al-Hussini H.S., Al-Rawahi A.Y., Al-Marhoon A.A., Al-Abri S.A., Al-Mahmooli I.H., Al-Sadi A.M., Velazhahan R. (2019). Biological control of damping-off of tomato caused by *Pythium aphanidermatum* by using native antagonistic rhizobacteria isolated from Omani soil. J. Plant Pathol..

[38] Al-Alawi A.K.S., Al-Mandhari A.A.S., Al-Mahmooli I.H., Al-Harrasi M.M.A., Al-Bulushi I.M., Al-Sadi A.M., Velazhahan R. (2023). Assessment of aflatoxin B1 content and aflatoxigenic molds in imported food commodities in Muscat, Oman. J. Agric. Mar. Sci..

[39] Vijayasamundeeswari A., Vijayanandraj S., Paranidharan V., Samiyappan R., Velazhahan R. (2010). Integrated management of aflatoxin B1 contamination of groundnut (*Arachis hypogaea* L.) with *Burkholderia* sp. and zimmu (*Allium sativum* L.× *Allium cepa* L.) intercropping. J. Plant Interact..

[40] Bozzola J.J., Russell L.D. (1999). Electron Microscopy: Principles and Techniques for Biologists.

[41] Al-Rahbi B.A.A., Al-Sadi A.M., Al-Harrasi M.M.A., Al-Sabahi J.N., Al-Mahmooli I.H., Blackburn D., Velazhahan R. (2023). Effectiveness of endophytic and rhizosphere bacteria from *Moringa* spp. in controlling *Pythium aphanidermatum* damping-off of cabbage. Plants.

[42] Jayakumar V., Sundar A.R., Viswanathan R. (2021). Biocontrol of *Colletotrichum falcatum* with volatile metabolites produced by endophytic bacteria and profiling VOCs by headspace SPME coupled with GC-MS. Sugar Tech..

[43] Al-Rashdi A., Al-Sadi A.M., Al-Harrasi M.M.A., Al-Sabahi J.N., Janke R., Velazhahan R. (2023). The effect of NaCl on growth and volatile metabolites produced by antagonistic endophytic bacteria isolated from *Prosopis cineraria*. Australas. Plant Pathol..

[44] Jard G., Liboz T., Mathieu F., Guyonvarc’h A., Lebrihi A. (2011). Review of mycotoxin reduction in food and feed: From prevention in the field to detoxification by adsorption or transformation. Food Addit. Contam. Part A.

[45] Ismail A., Gonçalves B.L., de Neeff D.V., Ponzilacqua B., Coppa C.F.S.C., Hintzsche H., Sajid M., Cruz A.G., Corassin C.H., Oliveira C.A.F. (2018). Aflatoxin in foodstuffs: Occurrence and recent advances in decontamination. Food Res. Int..

[46] Khan N., Martínez-Hidalgo P., Ice T.A., Maymon M., Humm E.A., Nejat N., Sanders E.R., Kaplan D., Hirsch A.M. (2018). Antifungal activity of *Bacillus* species against *Fusarium* and analysis of the potential mechanisms used in biocontrol. Front. Microbiol..

[47] Berrada I., Benkhemmar O., Swings J., Bendaou N., Amar M. (2012). Selection of halophilic bacteria for biological control of tomato gray mould caused by *Botrytis cinerea*. Phytopathol. Mediterr..

[48] Manikandan P., Moopantakath J., Imchen M., Kumavath R., SenthilKumar P.K. (2021). Identification of multi-potent protein subtilisin A from halophilic bacterium *Bacillus firmus* VE2. Microb. Pathog..

[49] Wang S., Huang Z., Wan Q., Feng S., Xie X., Zhang R., Zhang Z. (2020). Comparative genomic and metabolomic analyses of two *Pseudomonas aeruginosa* strains with different antifungal activities. Front. Microbiol..

[50] Huang Y.H., Wang X.J., Zhang F., Huo X.B., Fu R.S., Liu J.J., Sun W.B., Kang D.M., Jing X. (2013). The identification of a bacterial strain BGI-1 isolated from the intestinal flora of *Blattella germanica*, and its anti-entomopathogenic fungi activity. J. Econ. Entomol..

[51] Amer A., Hamdy B., Mahmoud D., Elanany M., Rady M., Alahmadi T., Alharbi S., AlAshaal S. (2021). Antagonistic activity of bacteria isolated from the *Periplaneta americana* L. gut against some multidrug-resistant human pathogens. Antibiotics.

[52] Shehabeldine A.M., Hashem A.H., Hasaballah A.I. (2022). Antagonistic effect of gut microbiota of the Egyptian honeybees, *Apis mellifera* L. against the etiological agent of Stonebrood disease. Int. J. Trop. Insect Sci..

[53] Miller D.L., Smith E.A., Newton I.L.G. (2021). A bacterial symbiont protects honey bees from fungal disease. mBio.

[54] Kotze C., Van Niekerk J., Mostert L., Halleen F., Fourie P. (2011). Evaluation of biocontrol agents for grapevine pruning wound protection against trunk pathogen infection. Phytopathol. Mediterr..

[55] De Boer W., Wagenaar A.M., Klein Gunnewiek P.J.A., van Veen J.A. (2007). In vitro suppression of fungi caused by combinations of apparently non-antagonistic soil bacteria. FEMS Microbiol. Ecol..

[56] Al-Daghari D.S.S., Al-Sadi A.M., Al-Harrasi M.M.A., Al-Sabahi J.N., Janke R., Velazhahan R. (2023). Antagonistic bacterial strains isolated from cabbage rhizosphere release antimicrobial volatile organic compounds against *Pythium aphanidermatum*. J. Agric. Mar. Sci..

[57] Halo B.A., Al-Yahyai R.A., Al-Sadi A.M. (2018). *Aspergillus terreus* inhibits growth and induces morphological abnormalities in *Pythium aphanidermatum* and suppresses *Pythium*-induced damping-off of cucumber. Front. Microbiol..

[58] Garg H., Li H., Sivasithamparam K., Kuo J., Barbetti M.J. (2010). The infection processes of *Sclerotinia sclerotiorum* in cotyledon tissue of a resistant and a susceptible genotype of *Brassica napus*. Ann. Bot..

[59] Elaasser M., El Kassas R. (2011). Detoxification of aflatoxin B1 by certain bacterial species isolated from Egyptian soil. World Mycotoxin J..

[60] Ali S., Hassan M., Essam T., Ibrahim M.A., Al-Amry K. (2021). Biodegradation of aflatoxin by bacterial species isolated from poultry farms. Toxicon.

[61] Gao X., Ma Q., Zhao L., Lei Y., Shan Y., Ji C. (2011). Isolation of *Bacillus subtilis*: Screening for aflatoxins B1, M1, and G1 detoxification. Eur. Food Res. Technol..

[62] Goncalves B.L., Rosim R.E., de Oliveira C.A.F., Corassin C.H. (2015). The in vitro ability of different *Saccharomyces cerevisiae*–based products to bind aflatoxin B1. Food Control.

[63] Peltonen K., El-Nezami H., Haskard C., Ahokas J., Salminen S. (2001). Aflatoxin B1 binding by dairy strains of lactic acid bacteria and bifidobacteria. J. Dairy Sci..

[64] El Khoury A., Atoui A., Yaghi J. (2011). Analysis of aflatoxin M1 in milk and yogurt and AFM1 reduction by lactic acid bacteria used in Lebanese industry. Food Control.

[65] Ahlberg S.H., Joutsjoki V., Korhonen H.J. (2015). Potential of lactic acid bacteria in aflatoxin risk mitigation. Int. J. Food Microbiol..

[66] Wu J., Wang Z., An W., Gao B., Li C., Han B., Tao H., Wang J., Wang X., Li H. (2024). *Bacillus subtilis* simultaneously detoxified Aflatoxin B1 and zearalenone. Appl. Sci..

[67] Siahmoshteh F., Siciliano I., Banani H., Hamidi-Esfahani Z., Razzaghi-Abyaneh M., Gullino M.L., Spadaro D. (2017). Efficacy of *Bacillus subtilis* and *Bacillus amyloliquefaciens* in the control of *Aspergillus parasiticus* growth and aflatoxins production on pistachio. Int. J. Food Microbiol..

[68] Campos-Avelar I., Colas De La Noue A., Durand N., Cazals G., Martinez V., Strub C., Fontana A., Schorr-Galindo S. (2021). *Aspergillus flavus* growth inhibition and aflatoxin B1 decontamination by *Streptomyces* isolates and their metabolites. Toxins.

[69] El-Nezami H., Kankaanpaa P., Salminen S., Ahokas J. (1998). Ability of dairy strains of lactic acid bacteria to bind a common food carcinogen, aflatoxin B1. Food Chem. Toxicol..

[70] Guan S., Ji C., Zhou T., Li J., Ma Q., Niu T. (2008). Aflatoxin B1 degradation by *Stenotrophomonas maltophilia* and other microbes selected using coumarin medium. Int. J. Mol. Sci..

[71] El-Nezami H., Kankaanpaa P., Salminen S., Ahokas J. (1998). Physicochemical alterations enhance the ability of dairy strains of lactic acid bacteria to remove aflatoxin from contaminated media. J. Food. Prot..

[72] Kim S., Lee H., Lee S., Lee J., Ha J., Choi Y., Yoon Y., Choi K.H. (2017). Microbe-mediated aflatoxin decontamination of dairy products and feeds. J. Dairy Sci..

[73] Xu L., Eisa Ahmed M.F., Sangare L., Zhao Y., Selvaraj J.N., Xing F., Wang Y., Yang H., Liu Y. (2017). Novel aflatoxin-degrading enzyme from *Bacillus shackletonii* L7. Toxins.

[74] Liu D.L., Yao D.S., Liang R., Ma L., Cheng W.Q., Gu L.Q. (1998). Detoxification of aflatoxin B1 by enzymes isolated from *Armillariella tabescens*. Food Chem. Toxicol..

[75] Cao H., Liu D., Mo X., Xie C., Yao D. (2011). A fungal enzyme with the ability of aflatoxin B1 conversion: Purification and ESI-MS/MS identification. Microbiol. Res..

[76] Shu X., Wang Y., Zhou Q., Li M., Hu H., Ma Y., Chen X., Ni J., Zhao W., Huang S. (2018). Biological degradation of aflatoxin B1 by cell-free extracts of *Bacillus velezensis* DY3108 with broad pH stability and excellent thermostability. Toxins.

[77] Lee L.S., Stanley J.B., Cucullu A.F., Pons W.A., Goldblatt L.A. (1974). Ammoniation of aflatoxin B1: Isolation and identification of the major reaction product. J. Assoc. Off. Anal. Chem..

[78] Cucullu A.F., Lee L.S., Pons Jr W.A., Stanley J.B. (1976). Ammoniation of aflatoxin B1. Isolation and characterization of a product with molecular weight 206. J. Agric. Food Chem..

[79] Mendez-Albores A., Nicolas-Vazquez I., Miranda-Ruvalcaba R., Moreno-Martinez E. (2008). Mass spectrometry/mass spectrometry study on the degradation of B-aflatoxins in maize with aqueous citric acid. Am. J. Agric. Biol. Sci..

[80] Iram W., Anjum T., Iqbal M., Ghaffar A., Abbas M., Khan A.M. (2016). Structural analysis and biological toxicity of aflatoxins B1 and B2 degradation products following detoxification by *Ocimum basilicum* and *Cassia fistula* aqueous extracts. Front. Microbiol..

[81] Wang L., Wu J., Liu Z., Shi Y., Liu J., Xu X., Hao S., Mu P., Deng F., Deng Y. (2019). Aflatoxin B1 degradation and detoxification by *Escherichia coli* CG1061 isolated from chicken cecum. Front. Pharmacol..

[82] Al-Owaisi A., Al-Sadi A.M., Al-Sabahi J.N., Sathish Babu S.P., Al-Harrasi M.M.A., Al-Mahmooli I.H., Abdel-Jalil R., Velazhahan R. (2022). In vitro detoxification of aflatoxin B1 by aqueous extracts of medicinal herbs. All Life.

[83] Huang J.S., Peng Y.H., Chung K.R., Huang J.W. (2018). Suppressive efficacy of volatile compounds produced by *Bacillus mycoides* on damping-off pathogens of cabbage seedlings. J. Agric. Sci..

[84] Gong A.D., Dong F.Y., Hu M.J., Kong X.W., Wei F.F., Gong S.J., Zhang Y.M., Zhang J.B., Wu A.B., Liao Y.C. (2019). Antifungal activity of volatile emitted from *Enterobacter asburiae* Vt-7 against *Aspergillus flavus* and aflatoxins in peanuts during storage. Food Control.

[85] Ye X., Chen Y., Ma S., Yuan T., Wu Y., Li Y., Zhao Y., Chen S., Zhang Y., Li L. (2020). Biocidal effects of volatile organic compounds produced by the myxobacterium *Corrallococcus* sp. EGB against fungal phytopathogens. Food Microbiol..

[86] Al-Rashdi A., Al-Hinai F.S., Al-Harrasi M.M.A., Al-Sabahi J.N., Al-Badi R.S., Al-Mahmooli I.H., Al-Sadi A.M., Velazhahan R. (2023). The potential of endophytic bacteria from *Prosopis cineraria* for the control of *Pythium aphanidermatum*-induced damping-off in cucumber under saline water irrigation. J. Plant Pathol..

[87] Rani A., Rana A., Dhaka R.K., Singh A.P., Chahar M., Singh S., Nain L., Singh K.P., Minz D. (2023). Bacterial volatile organic compounds as biopesticides, growth promoters and plant-defense elicitors: Current understanding and future scope. Biotechnol. Adv..

[88] Giorgio A., De Stradis A., Lo Cantore P., Iacobellis N.S. (2015). Biocide effects of volatile organic compounds produced by potential biocontrol rhizobacteria on *Sclerotinia sclerotiorum*. Front. Microbiol..

[89] Tyagi S., Lee K.J., Shukla P., Chae J.C. (2020). Dimethyl disulfide exerts antifungal activity against *Sclerotinia minor* by damaging its membrane and induces systemic resistance in host plants. Sci. Rep..

[90] Strobel G.A., Spang S., Kluck K., Hess W.M., Sears J., Livinghouse T. (2008). Synergism among volatile organic compounds resulting in increased antibiosis in *Oidium* sp.. FEMS Microbiol. Lett..

[91] Koilybayeva M., Shynykul Z., Ustenova G., Waleron K., Jońca J., Mustafina K., Amirkhanova A., Koloskova Y., Bayaliyeva R., Akhayeva T. (2023). Gas chromatography–mass spectrometry profiling of volatile metabolites produced by some *Bacillus* spp. and evaluation of their antibacterial and antibiotic activities. Molecules.

[92] Li X., Wang X., Shi X., Wang B., Li M., Wang Q., Zhang S. (2020). Antifungal effect of volatile organic compounds from *Bacillus velezensis* CT32 against *Verticillium dahliae* and *Fusarium oxysporum*. Processes.

[93] Gao H., Li P., Xu X., Zeng Q., Guan W. (2018). Research on volatile organic compounds from *Bacillus subtilis* CF-3: Biocontrol effects on fruit fungal pathogens and dynamic changes during fermentation. Front. Microbiol..

[94] Sholkamy E.N., Palsamy S., Raja S.S.S., Alarjani K.M., Alharbi R.M., Abdel-Raouf N., Alsamhary K.I., Duraipandiyan V., Mangaladoss F., Apu E.H. (2023). GC-MS analysis and bioactivity of *Streptomyces* sp. nkm1 volatile metabolites against some phytopathogenic fungi. Braz. Arch. Biol. Technol..

